# Disruption of actin filaments induces mitochondrial Ca^2+ ^release to the cytoplasm and [Ca^2+^]_c _changes in *Arabidopsis *root hairs

**DOI:** 10.1186/1471-2229-10-53

**Published:** 2010-03-24

**Authors:** Yuqing Wang, Yingfang Zhu, Yu Ling, Haiyan Zhang, Peng Liu, František Baluška, Jozef Šamaj, Jinxing Lin, Qinli Wang

**Affiliations:** 1 Key Laboratory of Photosynthesis and Molecular Environmental Physiology, Institute of Botany, Chinese Academy of Sciences, Beijing 100093, China; 2 Graduate School of Chinese Academy of Sciences, Beijing 100049, China; 3 College of Agronomy and Biotechnology, China Agricultural University, Beijing 100193, China; 4 Institute of Cellular and Molecular Botany, University of Bonn, Department of Plant Cell Biology, Kirschallee 1, D-53115 Bonn, Germany; 5 Centre of the Region Hana for Biotechnological and Agricultural Research, Faculty of Science, Palacky University, 78301 Olomouc, Czech Republic

## Abstract

**Background:**

Mitochondria are dynamic organelles that move along actin filaments, and serve as calcium stores in plant cells. The positioning and dynamics of mitochondria depend on membrane-cytoskeleton interactions, but it is not clear whether microfilament cytoskeleton has a direct effect on mitochondrial function and Ca^2+ ^storage. Therefore, we designed a series of experiments to clarify the effects of actin filaments on mitochondrial Ca^2+ ^storage, cytoplasmic Ca^2+ ^concentration ([Ca^2+^]_c_), and the interaction between mitochondrial Ca^2+ ^and cytoplasmic Ca^2+ ^in *Arabidopsis *root hairs.

**Results:**

In this study, we found that treatments with latrunculin B (Lat-B) and jasplakinolide (Jas), which depolymerize and polymerize actin filaments respectively, decreased membrane potential and Ca^2+ ^stores in the mitochondria of *Arabidopsis *root hairs. Simultaneously, these treatments induced an instantaneous increase of cytoplasmic Ca^2+^, followed by a continuous decrease. All of these effects were inhibited by pretreatment with cyclosporin A (Cs A), a representative blocker of the mitochondrial permeability transition pore (mPTP). Moreover, we found there was a Ca^2+ ^concentration gradient in mitochondria from the tip to the base of the root hair, and this gradient could be disrupted by actin-acting drugs.

**Conclusions:**

Based on these results, we concluded that the disruption of actin filaments caused by Lat-B or Jas promoted irreversible opening of the mPTP, resulting in mitochondrial Ca^2+ ^release into the cytoplasm, and consequent changes in [Ca^2+^]_c_. We suggest that normal polymerization and depolymerization of actin filaments are essential for mitochondrial Ca^2+ ^storage in root hairs.

## Background

The actin cytoskeleton is a dynamic structure that participates in many cellular functions including the maintenance of cell polarity and morphology, intracellular trafficking of organelles, cell motility, and cell division [1-4]. Light, touch, heat, hormones, pathogen attack, and many other extracellular stimuli lead to rapid structural changes of the actin cytoskeleton in plant cells [5,6]. Actin rearrangements in response to physiological cues have been described for root hairs responding to nodulation factors from *Rhizobia*, and for pollen tubes responding to self-incompatibility factors [7,8]. Increasing evidence suggests that actin filaments play an important role in cellular signal transduction [5,9].

Agents that act on actin to disrupt microfilaments dynamics have been used to investigate downstream reactions [10,11]. For example, latrunculins (Lat) and jasplakinolide (Jas) were used often in recent years to disrupt or stabilize actin filaments, which change the structure of the actin cytoskeleton immediately [11,12]. At the biochemical level, Lat have a straightforward and specific mode of action, which end up in a complete shift from filamentous actin (F-actin) to globular actin (G-actin) by forming G-actin-Lat complexes [12]. Jas stabilizes actin filaments and induces an apparent contraction of the actin cytoskeleton. In addition, Jas can disrupt actin filaments *in vivo *and induce the monomeric actin to polymerize into amorphous masses [13]. In this study, we also used latrunculin B (Lat-B) and Jas to disturb the normal dynamics of actin filaments.

Free Ca^2+ ^plays vital roles in cell development and is involved in a myriad of physiological functions. Cytoplasmic Ca^2+ ^is mainly from an influx of extracellular Ca^2+ ^through different plasma membrane cation channels [14,15]. Earlier investigations demonstrated that the cytoplasmic free Ca^2+ ^concentration, [Ca^2+^]_c_, is much lower than that outside the cell or in some inner membrane organelles [16]. In plants, this maintenance of low [Ca^2+^]_c _mainly depends on the cell wall or cellular Ca^2+ ^stores, such as vacuoles, the endoplasmic reticulum (ER), and mitochondria [17]. The regulation of Ca^2+ ^pools and the Ca^2+ ^cycle is critical for cells and has been well studied [18,19]. However, most research on plant cells has focused on vacuoles [20,21] and ER [22,23], and only a few studies have examined Ca^2+ ^in plant mitochondria [24].

Mitochondria are important Ca^2+ ^stores in animal and fungal cells [25,26]. They are constantly in motion, can undergo fission and fusion in response to cellular events, and can communicate with other Ca^2+ ^stores [27]. Mitochondria can move to and assemble in any cytoplasmic area where Ca^2+ ^levels are increasing or decreasing [28]. Mitochondria in plant cells move mainly along F-actin [29,30], and thus interactions between mitochondrial membranes and microfilaments affect the structure and positioning of mitochondria and direct their movements. Nevertheless, it is not known whether actin filaments have an impact on the function of mitochondria. Some recent reports proposed that F-actin regulates mitochondrial motion and fission [30,31]. Other studies have focused on Ca^2+ ^carriers and Ca^2+ ^channels of mitochondrial membranes in animal cells [32,33]. Few studies have considered the effect of the disruption of microfilaments on mitochondrial Ca^2+ ^transport across membranes [34]. Thus, it is necessary to investigate the interaction between F-actin and mitochondrial Ca^2+ ^storage.

Some research demonstrated that mitochondrial membrane potential (Ψ_m_) and mitochondrial permeability transition pore (mPTP) were also involved in Ca^2+ ^homeostasis in plant cells [35,36]. Ψ_m _has been used as a marker for mitochondrial activity [37,38]. Normally, active mitochondria are polarized and have a high positive potential [37]. The mPTP is a nonselective, high conductance channel with three main components, including the voltage-dependent anion channel (VDAC in the outer mitochondrial membrane), adenine nucleotide translocase (ANT in the inner mitochondrial membrane), and the matrix protein cyclophilin D [39]. The mPTP opening allows diffusion of solutes with a molecular mass up to about 1500 Da, and they can exist in two forms: reversible normal opening at low conductance and irreversible opening at high conductance [40]. Some previous studies have focused on the association between mitochondrial membrane potential and mPTP [41,42]. Normal mPTP opening was important for maintaining Ψ_m _in animal and filamentous fungal cells [41,43]. Studies on plant cells also showed that the mitochondrial membrane potential decreased when mPTP opened abnormally [39,44].

Tip-growing cells represent an ideal system in which to investigate the role of the actin cytoskeleton in signal transduction [9]. Root hairs and pollen tubes are the most familiar models for polarized tip growth. To investigate the effect of the disruption of microfilaments on mitochondrial and cytoplasmic Ca^2+^, we chose *Arabidopsis *root hairs as an experimental material. Specifically, we focused on changes in mitochondrial Ca^2+ ^concentration ([Ca^2+^]_m_), and its relationship with [Ca^2+^]_c_, which is assumed to be linked with and essential for root hair tip growth. The purpose of this study was to evaluate the effects of disruption of actin filaments on mitochondrial Ca^2+ ^storage, [Ca^2+^]_c_, and the interaction between mitochondrial Ca^2+ ^and cytoplasmic Ca^2+ ^in root hairs.

## Results

### Distribution and Ca^2+ ^concentration of mitochondria in root hairs

**Figure 1:**
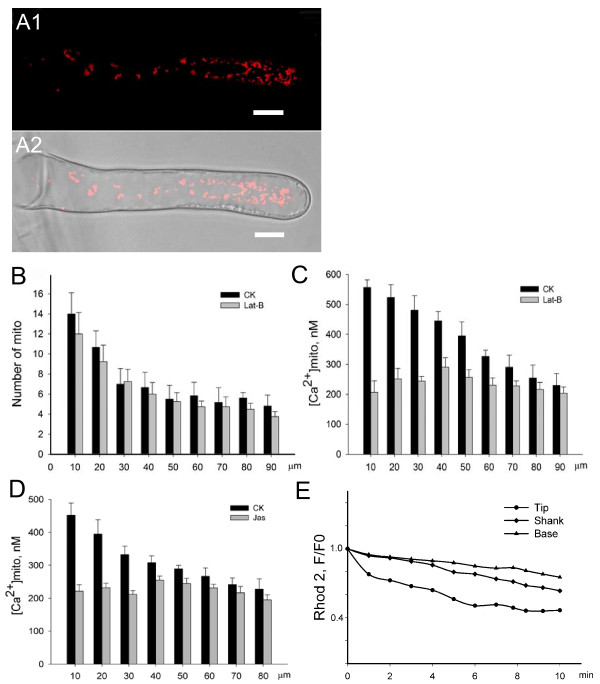
**The gradient of [Ca^2+^]_m _in root hairs was disrupted by Lat-B and Jas**. (A1) Fluorescence micrograph of mitochondria (red particles) labeled with Rhod-2 in a normally growing root hair. (A2) Superimposition of the bright field image and fluorescence micrograph of the same root hair. Scale bars = 10 μm. (B) The gradient distribution of mitochondria in root hairs did not obviously change after a 10-min treatment with 500 nM Lat-B. The graph depicted the number of mitochondria from a 10-μm distance from the apex to the base region at 10-μm intervals. CK represented the measured data before treatment. (C) The gradient of [Ca^2+^]_m _in a normal root hair was disrupted after a 3-min treatment with 500 nM Lat-B. The graph showed the concentration from a 10-μm distance from the apex to the base region at 10-μm intervals. CK represented the measured data in drug-free medium before treatment. (D) The gradient of [Ca^2+^]_m _was dissipated after 3 min of treatment with Jas. CK represented the measured data in drug-free medium before treatment. (E) Decreases in [Ca^2+^]_m _in the tip, shank, and base regions of the root hair after a 10-min treatment with 500 nM Lat-B. F0 represented the fluorescence intensity value of Rhod-2 before treatments, and F represented the fluorescence intensity value in same mitochondria after treatments.

In this study, fluorescent mitochondrial dyes, MitoTracker and Rhod-2, were used to visualize mitochondria and quantitate the mitochondrial Ca^2+ ^concentration. Both cable-like or dot structures dyes labeled were highly dynamic. In elongating root hairs, mitochondrial density was uneven and mitochondrial distribution showed a sub-tip to base gradient (Figure 1A and 1B). After root hairs were incubated in 10 μM Rhod-2, and [Ca^2+^]_m _was calculated based on the fluorescence density value of Rhod-2. In most of the mitochondria, [Ca^2+^]_m _varied from 230 nM to 800 nM, and in a small number, [Ca^2+^]_m _reached 1 μM. [Ca^2+^]_m _was also unequal in different areas. In addition, there was a calcium concentration gradient along the root hair long axis with [Ca^2+^] in the apical and subapical mitochondria being about twice that in mitochondria at the root hair base (Figure 1C).

When root hairs were treated with 500 nM Lat-B, the distribution pattern of mitochondria did not obviously change; the mitochondrial number remained the highest in the subapical region and lowest in the base of the root hair (Figure 1B). However, the calcium concentration gradient of mitochondria in different regions of the root hair disappeared after 3 min (Figure 1C). Treatment with 250 nM Jas also dramatically disrupted the [Ca^2+^]_m _gradient (Figure 1D). The rate and magnitude of [Ca^2+^] decrease in apical and subapical mitochondria were greater than those of mitochondria in other areas of the root hair in the Lat-B and Jas treatments, with the [Ca^2+^]_m _decrease in the root hair tip being about 50% greater than that in the root hair base in the Lat-B treatment (Figure 1E).

### Mitochondrial calcium release

**Figure 2:**
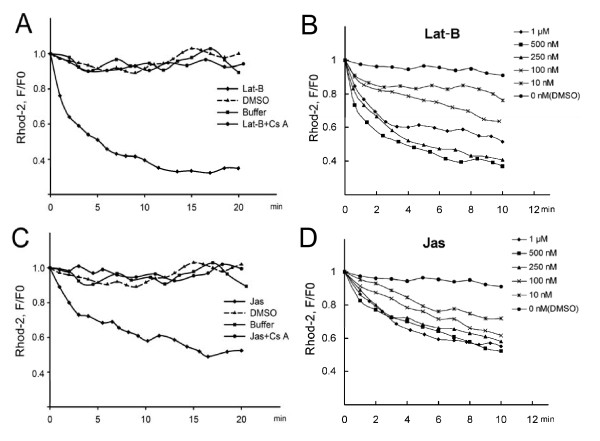
**Changes in [Ca^2+^]_m _induced by Lat-B and Jas treatments**. [Ca^2+^]_m _was assessed based on fluorescence intensity of Rhod-2, as described in the Methods section. (A) and (C) The diamond curve showed the decrease in [Ca^2+^]_m _induced by 500 nM actin drugs treatment with in 20 min; the triangle curve showed the DMSO vehicle control; the square curve represented the buffer control in drug-free medium; and the dotted curve showed the change in [Ca^2+^]_m _with 30-min pretreatment with 2 μM Cs A before actin drugs application. (B) and (D) The effects of actin drugs on [Ca^2+^]_m _at different concentrations. Root hairs were treated with 0 (DMSO), 10, 100, 250, 500 nM and 1 μM actin drugs for 10 min. The graphs showed the dose-dependent effect of actin drugs in the 10-500 nM concentration range on [Ca^2+^]_m _in the root hair. These data were from concentration changes of mitochondria in sub-tip area of the root hair. F0 represented the fluorescence intensity value of Rhod-2 before treatments, and F represented the fluorescence intensity value in same mitochondria after treatments.

In normally growing root hairs, [Ca^2+^]_m _was frequently higher than 230 nM. After treatment with 500 nM Lat-B, the mitochondrial Ca^2+ ^level decreased by 65% after 10 min (Figure 2A; see Additional File 1: Movie 1 for a original example of a series of images); with 1 μM Lat-B, the decrease was only 46% after 10 min (Figure 2B). At 10 nM Lat-B, there was a 22% decrease 10 min later (Figure 2B). The actin stabilizer Jas induced changes similar to those seen in the Lat-B treatments, but the rate of decrease in [Ca^2+^]_m _induced by Jas was slower than that with Lat-B. For example, 500 nM Jas decreased the [Ca^2+^]_m _to 52% of the control value after 10 min of treatment (Figure 2C). When treated with lower concentrations, from 250 nM to 10 nM Jas, the rate of decrease was gradually reduced. Thus, the effects of actin drugs were dose-dependent in the 10-500 nM concentration range, with a higher drug concentration inducing a greater and longer decrease in [Ca^2+^]_m _(Figure 2B and 2D).

Cs A is an mPTP inhibitor used in various cell types as it can bind specifically to the membrane protein cyclophilin D of the mPTP [45]. With a pretreatment of 2 μM Cs A for 30 min, [Ca^2+^]_m _did not change after treatment with actin drugs in comparison to that before treatment in at least six independent experiments (Figure 2A and 2C). Furthermore, the inhibitory effects of Cs A on Jas were similar to its effects on Lat-B. These results suggested that the mitochondrial Ca^2+ ^release induced by these drugs was prevented by Cs A.

### Decrease in mitochondrial membrane potential

**Figure 3:**
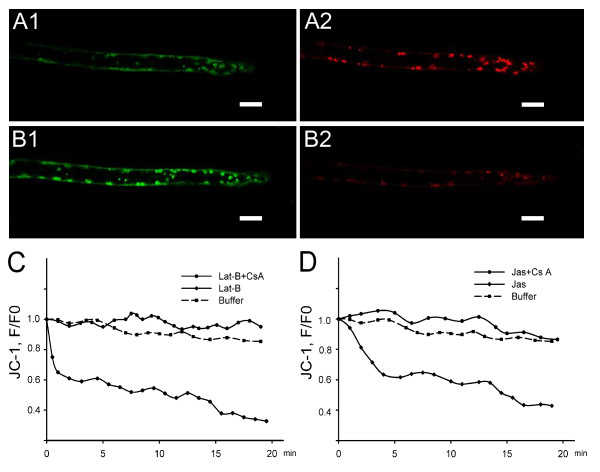
**Effects of Lat-B and Jas on mitochondrial membrane potential**. Mitochondrial membrane potential was assessed using the fluorescent potentiometric dye JC-1. Red particles represented mitochondria with high membrane potential, and green particles represented those with low membrane potential. (A1) Green fluorescence image of mitochondria with low membrane potential labeled with JC-1 in control root hair. (A2) Red fluorescence image of mitochondria with high membrane potential labeled with JC-1 in the same control root hair. (B1) and (B2) Green and red fluorescence images of mitochondria in the same root hair, 5 min after the addition of 500 nM Lat-B. (C) and (D) The actin-acting drugs reduced the red/green fluorescence ratio, i.e. mitochondrial membrane potential. This effect was inhibited by a 30-min pretreatment with 2 μM Cs A. The diamond curve showed the 500 nM actin drugs treatment over a 20 min period; the dotted curve showed the change in [Ca^2+^]_m _with Cs A pretreatment before actin drugs application; and the square curve represented the buffer control in drug-free medium. F represented the ratio of red/green fluorescence, and F0 represented the ratio of red/green fluorescence before treatments. All scale bars = 10 μm.

The vital dye JC-1 is a fluorescent cationic dye that has been used to investigate mitochondrial membrane potential [46]. Mitochondria with high membrane potential fluoresce red, while those with low potential fluoresce green at 488 nm laser excitation, indicating that mitochondria are polarized when shifting fluorescence emission from green to red [46]. In the control growing root hairs, most mitochondria fluoresced red (Figure 3A1 and 3A2). The application of 500 nM Lat-B caused immediate mitochondrial depolarization, inducing the majority of JC-1 to become green (Figure 3B1 and 3B2). The ratio of red/green fluorescence decreased to 65% of the control ratio after 1 min and to 33% after 20 min (Figure 3C). Treatment with 500 nM Jas caused a change similar to, but much slower than that of Lat-B treatments, with the red/green ratio decreasing to 62% of the control value after about 5 min and to 43% after 20 min (Figure 3D).

### mPTP opening induced by actin drugs

**Figure 4:**
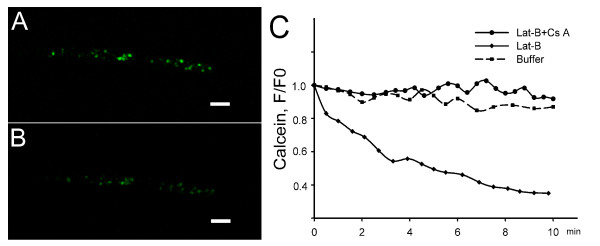
**Opening of the mPTP caused by Lat-B treatment, as visualized by reduced calcein fluorescence in mitochondria**. Root hair cells were loaded with calcein, and the green fluorescence represented mitochondria labeled with calcein in the root hairs. The data were representative of six independent experiments. (A) Fluorescence photograph of calcein trapped in the mitochondria in a control root hair. (B) Fluorescence image of calcein in the mitochondria after 5 min of treatment with 500 nM Lat-B in the same control root hair. (C) Changes in the fluorescence of trapped calcein over time. The diamond curve showed the decrease in calcein fluorescence induced by 500 nM Lat-B treatment in 10 min duration; the dotted curve showed the change with a 30-min pretreatment with 2 μM Cs A before Lat-B application; and the square curve represented the buffer control in drug-free medium. F0 represented the fluorescence intensity value of calcein before treatments, and F represented the fluorescence intensity value in same mitochondria after treatments. Scale bars = 10 μm.

The opening of mPTP was monitored by the calcein/Co^2+ ^imaging technique [47]. Co^2+ ^can quench calcein fluorescence in the cytoplasm but not that in the mitochondria; thus the decrease of calcein fluorescence in mitochondria indicated the reduction of mitochondrial Ca^2+ ^through open mPTP. In control root hairs, mitochondria exhibited green calcein fluorescence (Figure 4A). Treatment with 500 nM Lat-B induced a loss of calcein fluorescence from the mitochondria (Figure 4B), with a decrease to about 35% of the fluorescence density before treatment within 10 min (Figure 4C). This result indicated that Co^2+ ^entered the mitochondria after Lat-B treatment. This effect was prevented by pretreatment with the mPTP inhibitor Cs A (2 μM) (Figure 4C). The application of Jas showed similar effects to Lat-B treatments; the calcein fluorescence was reduced to 40% of the original value (data not shown).

### Changes in intracellular Ca^2+ ^concentration

**Figure 5:**
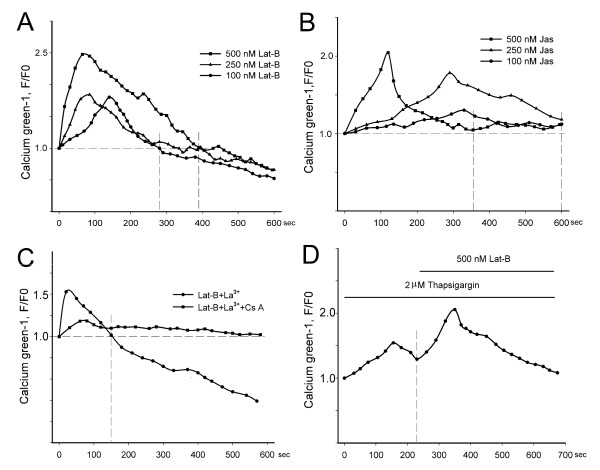
**Changes in cytoplasmic [Ca^2+^] induced by actin-acting drugs**. [Ca^2+^]_c _was assessed based on the fluorescence of Calcium green-1, as described in the Methods section. All data were representative of at least four independent experiments. F0 represented the fluorescence intensity value of Calcium green-1 before treatments, and F represented the fluorescence intensity value in same root hair after treatments. (A) and (B) Changes in [Ca^2+^]_c _after the application of actin drugs at different concentrations in testing medium over a 10 min period. The intersections of the horizontal dashed line and the two vertical dashed lines indicated when [Ca^2+^]_c _decreased to a level lower than that of before treatment. (C) The effect of 100 μM La^3+ ^on the Lat-B-induced [Ca^2+^]_c _increase as indicated by Calcium green-1 fluorescence intensity. The intersection of the horizontal and vertical dashed lines indicated when [Ca^2+^]_c _decreased to a level lower than that of before treatment. (D) Ca^2+ ^release from internal Ca^2+ ^stores due to the inhibition of the ER by 2 μM thapsigargin, after which Lat-B elicited a long lasting [Ca^2+^]_c _increase attributable to the opening of the mPTP. The drugs were applied for the durations indicated by the horizontal bars. The dashed line showed the start time of the Lat-B treatment.

The control root hair displayed a typical tip-focused cytoplasmic Ca^2+ ^gradient (see Additional File 2: Figure S1). When treated with 500 nM Lat-B, this gradient disappeared, i.e, [Ca^2+^]_c _showed a sharp but short elevation that lasted about 4-6 min and was followed by a continuous decrease (Figure 5A; Additional File 3: Figure S2). At lower concentrations (250 nM and 100 nM), Lat-B induced a smaller elevation of [Ca^2+^]_c _and shorter duration (Figure 5A). Compared to Lat-B, Jas treatments resulted in a slower and smaller elevation of [Ca^2+^]_c _that was not followed by a continuous reduction (Figure 5B). Interestingly, the Jas treatments with lower concentration induced [Ca^2+^]_c _elevation to a lesser extent but for a longer time (Figure 5B). Together, changes in [Ca^2+^]_c _and [Ca^2+^]_m _caused by actin drugs showed similar kinetics (Figure 5A and 5B, Figure 2A and 2C); the duration of the [Ca^2+^]_c _increase and simultaneous intense [Ca^2+^]_m _decrease were 4.5 ± 1.0 min and 5.0 ± 1.0 min, respectively.

La^3+ ^is a non-specific cation channel inhibitor that blocks plasma membrane Ca^2+^-permeable channels in plants. The sharp elevation in [Ca^2+^]_c _induced by Lat-B treatment was significantly weakened after extracellular Ca^2+ ^influx was blocked by the addition of 100 μM La^3+ ^(Figure 5C) or Ca^2+^-free solution (data not shown). This result indicated that Ca^2+ ^influx played a role in the [Ca^2+^]_c _elevation induced by Lat-B treatment.

Thapsigargin, a specific inhibitor of the ER Ca^2+^-ATPase, can increase cytoplasmic Ca^2+ ^levels [48]. In our experiments, treatment with thapsigargin depleted the ER Ca^2+ ^stores, as revealed by a slow [Ca^2+^]_c _increase in root hairs (Figure 5D). The subsequent addition of Lat-B produced a second lasting [Ca^2+^]_c _increase. In the presence of Cs A, thapsigargin evoked a similar transient change in [Ca^2+^]_c_, but the subsequent addition of Lat-B had no effect on [Ca^2+^]_c _(data not shown).

### Calcium flux at the root hairs surface

**Figure 6:**
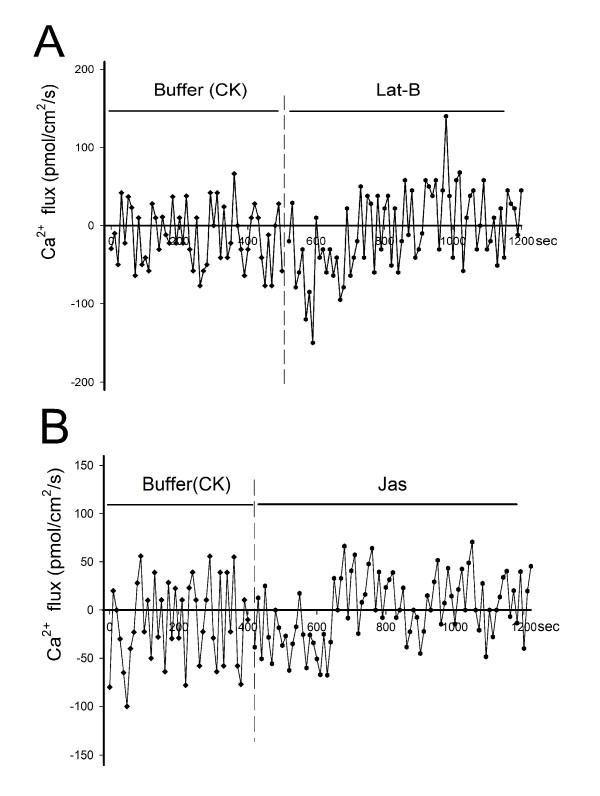
**Changes in cellular Ca^2+ ^influx and efflux measured in root hairs in response to actin-acting drugs treatments**. The data were obtained using non-invasive scanning ion-selective electrode measurements, which were taken at predefined locations (apex of root hair), with the Ca^2+ ^selective probe positioned 2 μm from the root hair surface. (A) Lat-B treatment induced a rapid increase in extracellular Ca^2+ ^influx, followed by obvious net efflux after 5 min. (B) Jas treatment induced a lasting increase in extracellular Ca^2+ ^influx within the first 5 min, followed by obvious net efflux. Positive values represent Ca^2+ ^efflux while negative values represent influx.

To clarify the reason for a decrease in [Ca^2+^]_c_, we used a scanning ion-selective electrode technique (SIET), to monitor Ca^2+ ^fluxes through the root hair surface (see Additional File 4: Figure S3). Control root hairs showed Ca^2+ ^influx and efflux at the cell surface, and Ca^2+ ^influx prevailed in the control hair apex (Figure 6A). This normal Ca^2+ ^flux was disrupted by Lat-B treatments; influx occurred within the first 5 min and an obvious efflux was noted after the primary influx (Figure 6A). The Ca^2+ ^influx intensity induced by Lat-B was 1.5-2 times that of the control, although the efflux intensity was not significantly changed. These results indicated a net Ca^2+ ^decrease from outward flux subsequently induced by microfilaments depolymerization. The change in Ca^2+ ^flux of cells after treatment with 250 nM Jas was similar to that in the Lat-B treatment. The obvious Ca^2+ ^influx lasted about 4-5 min initially, at an intensity equal to that of the control influx, and was followed by a lasting outward Ca^2+ ^flux (Figure 6B). These results were consistent with the results described above for changes in [Ca^2+^]_c _induced by Lat-B and Jas.

### Mitochondria, ER, and microfilaments showed co-localization

**Figure 7:**
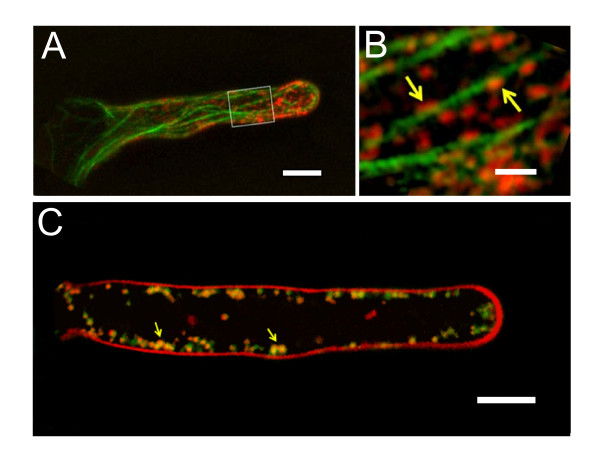
**Co-visualization of microfilaments, mitochondria, and endoplasmic reticulum**. (A) Root hair transformed with *35S::GFP-FABD2 *and stained with MitoTracker. Mitochondria and microfilaments were pseudo-colored red and green, respectively. Note the distribution of mitochondria along actin filaments and bundles. (B) Higher magnification of the area highlighted in (A). The arrows pointed to mitochondria anchored to microfilaments. (C) Root hair transformed with *35S::GFP5-ER *and stained with MitoTracker. Mitochondria and ER were pseudo-colored red and green, respectively. The overlap was showed in yellow. Scale bars: (A and C), 10 μm; (B), 1.5 μm.

To further verify possible interactions among mitochondria, ER, and microfilaments, these cellular components were co-visualized under a Zeiss confocal microscope. As shown in Figure 7, mitochondria were distributed mostly along actin filaments in the root hairs (Figure 7A and 7B). When the co-localization of mitochondria and ER was observed, ER with green fluorescence overlapped mitochondria with red fluorescence, and the area of overlap was yellow after the two channel images were merged. It was evident that there was co-localization between mitochondria and ER in root hairs (Figure 7C). Based on these results, we speculated that the mitochondria, ER, and microfilaments were partly co-localized. After microfilament disruption by Lat-B, the degree of overlap between the ER and mitochondria decreased from 66% to 45%; microfilament stabilization induced a similar change in overlap, from 80% to 55%.

## Discussion

Mitochondria are important Ca^2+ ^stores and move mainly along F-actin in plant cells [29,49]. Thus, interactions with microfilaments may affect the positioning and properties of mitochondria. Recent reports showed that F-actin regulated mitochondrial motion as well as fission and fusion [30,38]. Yet there were few reports on the effects of the cytoskeleton on mitochondrial Ca^2+ ^stores, or regulation of these stores. Mironov et al. demonstrated in mouse neurons that structural changes in microtubules modulated Ca^2+ ^release from mitochondria and ER [50]. However, to our knowledge, there are no reports on the regulation of mitochondrial calcium by the cytoskeleton in plant cells. In the present study, we observed that the pharmacological disruption of actin filaments affected not only Ca^2+ ^influx and efflux in root hairs but also mitochondrial Ca^2+ ^release, whereas actin polymerization induced a slower Ca^2+ ^decrease compare with F-actin depolymerization. These data suggested a novel role of actin filaments in cellular Ca^2+ ^homeostasis in plant cells.

We found that mitochondrial Ca^2+ ^release was non-uniform. The release of Ca^2+ ^from mitochondria in the tip and sub-tip of root hairs occurred faster than that in the shank and base. We also demonstrated an uneven and polarized distribution of mitochondria in root hairs, where the quantity of mitochondria decreased gradually from sub-tip to base. This was to some extent similar to the tip-to-base polarized distribution in other polarized cells such as neurons and fungal cells [51,52]. Furthermore, our results revealed a concentration gradient of mitochondrial Ca^2+^, from tip to base of the root hairs, similar to the cytoplasmic Ca^2+ ^gradient in the root hairs. Based on the present results and the research on motion velocity of mitochondria in *Arabidopsis *root hairs [30], we speculated that owing to mitochondria in the apical and subapical region moved the slowest, they could absorb or release more Ca^2+ ^in the tip with high [Ca^2+^]_c _of the root hairs. Those mitochondria in the middle and base of the root hairs moved very fast in the low [Ca^2+^]_c _surroundings, and maintained a lower [Ca^2+^]_m _than mitochondria in the tip.

Mitochondrial [Ca^2+^] was heterogeneous in cells. Collins et al. reported that mitochondria in the periphery of Hela cells sequestered more Ca^2+ ^than those located in the perinuclear region when [Ca^2+^]_c _increased rapidly [53]. The heterogeneity of mitochondrial [Ca^2+^] has not been previously investigated in plant cells. We report here for the first time that the rate of Ca^2+ ^release from mitochondria decreased gradually from the tip to the base in *Arabidopsis *root hairs, although we cannot explain the precise mechanism of this phenomenon. The most probable explanation is that the F-actin anchoring mitochondria in the tip of root hairs consisted mostly of fine and short actin filaments [54,55]; and was depolarized or polarized more quickly than the thick actin bundles in the shank or the base of the root hairs. Thus actin may act more rapidly on the outer membrane of mitochondria at the root hair tip. Another possibility is that mitochondria with high Ca^2+ ^concentrations or those located in cellular microdomains with high [Ca^2+^]_c _could more quickly efflux Ca^2+ ^than mitochondria with low Ca^2+ ^concentrations or those located in other cellular microdomains with low [Ca^2+^]_c_. Thus, we conclude that the uneven Ca^2+ ^release from mitochondria in root hairs resulted mainly from the heterogeneous distribution of F-actin and uneven [Ca^2+^] of mitochondria and cytoplasm in polarized root hairs.

Mitochondrial membrane potential has often been used as a marker to determine mitochondrial activity, owing to the fact that normal active mitochondria are polarized with a high positive potential [37,38]. Some studies have already investigated the relationship between F-actin and Ψ_m _in yeast and mammalian cells [56,57]. In yeast, a decrease in actin dynamics in certain actin mutants significantly reduced the mitochondrial membrane potential [58]. In cultured neurons, a deficiency of the actin depolymerizing protein gelsolin promoted the loss of Ψ_m _because of a reduction in actin dynamics [59]. Foger et al. found that treatment of T cells with Lat-A or Jas increased or decreased mitochondrial membrane potential, respectively [60]. Our data on plant root hairs showed that both Lat-B and Jas treatments caused a Ψ_m _decrease. This discrepancy may be attributable to the use of different materials in the two experiments. Foger et al. used coronin-defective T cells [60], which have a higher F-actin content, thus Lat-A would increase rather than decrease actin dynamics in the control T cells. Therefore, it seems reasonable to conclude that reduced actin dynamics could decrease mitochondrial membrane potential.

Some previous studies have focused on the association between mPTP and the cytoskeleton. Microtubule-acting drugs induced irreversible mPTP opening in mouse neurons [50]. Early investigations demonstrated that microtubule drugs suppressed mPTP closure after overfull Ca^2+^-induced mPTP opening [61]. Xu et al. found that actin could modulate the gating of VDAC in Neurospora crassa [43]. In the present study, we found that disruption of microfilaments caused by actin-acting drugs induce irreversible mPTP opening. Given that the VDAC forms a complex with ANT in the mitochondria and interact directly with actin filaments, we speculated that conformational changes in actin filaments caused by Lat-B and Jas promote the interaction between VDAC and ANT by imposing mechanical stress on VDAC and subsequently lead to the opening of mPTP at high conductance. Indeed, a similar conclusion was reported in a study on the structural change of microtubules induced the irreversible mPTP opening in mouse neurons [50].

Previous studies reported that the disruption of actin filaments and microtubules caused an increase or decrease in [Ca^2+^]_c_. In rat neurons, actin depolymerization and polymerization attenuated and enhanced, respectively, the increase in [Ca^2+^]_c _resulting from IP3-mediated Ca^2+ ^release [62]. In another study, disruption of the actin cytoskeleton with Cyto-D enhanced the parathyroid hormone (PTH) induced increase of intracellular Ca^2+^, whereas stabilization of actin with phalloidin prevented PTH-enhanced [Ca^2+^]_i _elevation in osteoblasts [63]. Some studies showed that actin filaments can modify Ca^2+ ^influx through plasma membrane Ca^2+ ^channels in different plant cells including pollen tubes [55,64]. Wang et al. found that actin depolymerization reagents significantly increased cytoplasmic Ca^2+ ^levels by increasing inward Ca^2+ ^flux through hyperpolarization-activated Ca^2+ ^permeable channels in pollen protoplasts and pollen tubes [55]. In the present study, we found that both actin depolymerization and polymerization induced [Ca^2+^]_c _elevation, followed by a decrease, accompanied by mitochondrial Ca^2+ ^release. These results differed from conclusions based on investigations in pollen tubes and osteoblasts, where Lat-A and Cyto-D only increased [Ca^2+^]_c _[55,63]. This difference may be attributable to two factors: the previous studies did not examine the condition with no extracellular Ca^2+^, and experimental materials were based on pollen protoplasts instead of intact root hair cells. Except for the interaction with cation channels, actin filaments also possibly interacted with Ca^2+^-ATPases, G-protein and Ca^2+^-binding proteins and then induced the [Ca^2+^]_c _elevation [65-67]. In addition, there was a possibility that mitochondria transport superoxide anions to the cytosol after mPTP opening and activate ROS-activated Ca^2+^-permeable channels in the plasma membrane [68,69].

Our results showed that mitochondrial Ca^2+ ^release and extracellular Ca^2+ ^influx caused an increase in [Ca^2+^]_c_, and the subsequent intracellular Ca^2+ ^efflux induced a decrease in [Ca^2+^]_c_. This conclusion was based on the following analysis: the sharp elevation in [Ca^2+^]_c _was weakened after blocking of Ca^2+ ^influx, probably indicating that Ca^2+ ^influx is required for the increase in [Ca^2+^]_c _except for mitochondrial Ca^2+ ^release; and disruption of actin filaments induces the release of Ca^2+ ^from mitochondria, which increased Ca^2+ ^further activated the Ca^2+^-induced Ca^2+ ^release (CICR) from the ER [50,70], accompanied by extracellular Ca^2+ ^influx.

## Conclusion

**Figure 8:**
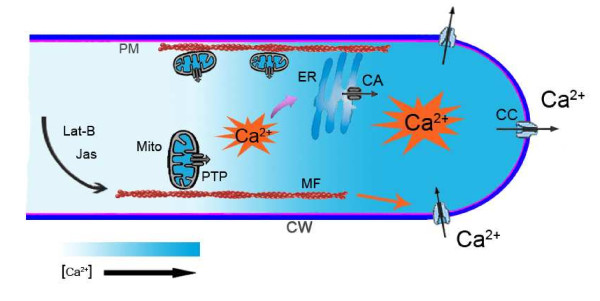
**Interactions between mitochondria, endoplasmic reticulum, and actin filaments**. Mitochondria-bound microfilaments are depolymerized by Lat-B and are stabilized by Jas, and either change in the actin structure promotes the opening of mPTP. Because of the close apposition of mitochondria to ER, Ca^2+ ^released from mitochondria can activate the Ca^2+^-induced Ca^2+ ^release (CICR) mechanism in ER, which triggers spontaneous [Ca^2+^]_c _spikes. The disruption of actin filaments also induces an increase in Ca^2+ ^influx at the root hair surface. The signal of Ca^2+ ^increase in the cytoplasm triggers final Ca^2+ ^efflux. Mito, mitochondria; ER, endoplasmic reticulum; MF, microfilaments; CW, cell wall; PM, protoplasm membrane; PTP, permeability transition pore; CA, Ca^2+^-ATPase, CC, Ca^2+^-channel.

In summary, our results indicate that disruption of actin filaments promoted an interaction between mitochondrial Ca^2+ ^and cytoplasmic Ca^2+^, as generalized in our model (Figure 8). Our data provided new functional evidence that the actin cytoskeleton plays an important and specific role in maintaining the mitochondrial Ca^2+ ^buffer function by regulating the opening of the mPTP and the positioning of mitochondria close to the ER. This study has three main findings: (1) Lat-B and Jas depolarized mitochondrial membranes and induced Ca^2+ ^release from mitochondria via the mPTP; (2) mitochondria displayed a [Ca^2+^] gradient from tip to base in living root hairs; and (3) actin-acting drugs first induced an increase followed by a decrease in [Ca^2+^]_c_. Based on these results, we conclude that the disruption of actin filaments induced the release of Ca^2+ ^from mitochondria to the cytoplasm with an increase in [Ca^2+^]_c_; these increased cytoplasmic Ca^2+ ^further induced the release of Ca^2+ ^from the ER and caused changes in Ca^2+ ^flux in *Arabidopsis *root hairs.

## Methods

### Plant materials

Seeds of *Arabidopsis thaliana *(Columbia 0) were surface sterilized, germinated on 1/2 MS medium supplemented with 0.1% (w/v) sucrose and 0.7% (w/v) plant agar, and grown at 22°C with a light/dark cycle of 16/8 h. The seedlings grew for 3-4 days and were chosen for experiments when roots were 1-2 cm long.

### Organelles and Ca^2+ ^labeling

All dyes were from Invitrogen (Carlsbad, CA, USA). Plants were incubated at 4°C in the dark for 30 min with one of the following dyes: 10 μM Rhod-2/AM (Rhod-2), 2 μg/ml JC-1, and 1 μM calcein/AM (calcein); or for 1 h with 1 μM Fluo-4/AM, or for 15 min with 100 nM MitoTracker. The incubation with Fluo-4/AM was at 4°C and followed the method of Zhang [71]. Mitochondria and ER were co-visualized using MitoTracker and the *35S::mGFP5-ER *construct in transformed plants. Mitochondria and microfilaments were co-localized using MitoTracker and the *35S::GFP-FABD2 *construct in transformed plants, respectively. After the plants were loaded with dyes, they were kept in dye-free medium for at least 30 min before starting the experiments.

### Confocal Microscopy

The root hairs were viewed under a 63× water immersion objective. When scanning the fluorescence of mitochondria, we used Z-stacks scanning to detect the root hairs with normal streaming. Before treatment, the position of an individual slide was fixed and noted in the object stage, the buffer below the cover glass was absorbed with thin filter paper and the drugs were added gently with a 10 μL micropipettor. Images were collected before and after treatment respectively.

Images obtained using a confocal laser microscope (Zeiss 510 Meta, Jena, Germany) were digitized at 8-bit resolution and analyzed using LSM 5 Image Browser or Image J software. Digital images were acquired every 5-10 s, and the average fluorescence intensities of all relevant regions were recorded and stored for subsequent analysis. Wide field fluorescence images were collected with exposure times of between 100 ms and 300 ms. The fluorescence of Fluo-4, Calcium green-1, calcein, and JC-1 were imaged using a 488 nm laser line for excitation, and the fluorescence was isolated using a dichroic mirror with mid-reflection at 505 nm and an emission filter (535 ± 15 nm). For Rhod-2, the emission was collected using a 535 nm primary dichroic mirror and the Meta-detector of the microscope.

### Fluorescence measurements

The fluorescence patterns of Rhod-2 and JC-1 in root hairs showed punctuate structures representing individual mitochondria or their aggregates. Fluorescence intensity was measured in 1 μm-diameter regions of interest encircling the bright spots corresponding to mitochondria. The signals from 4-6 mitochondria within a cell were averaged. These observations were repeated 6-8 times in different *Arabidopsis *plants, i.e. 6-8 root hairs selected randomly. Except for the analysis of density and concentration differences of the mitochondria from different regions of the root hairs, other data on mitochondria were obtained from the whole root hair rather than from certain specific region of the root hair. Before each experiment, root hair morphology and vitality were observed using transmitted light to ensure that normal cytoplasmic streaming was present in the growing root hairs. Each test was repeated at least three times for each root hair developmental period.

### Quantification of calcium concentrations

The Ca^2+^-sensitive fluorescent probes Fluo-4/AM ester and Calcium green-1-10-kDa dextran were used to measure [Ca^2+^]_c _in the root hairs. The latter Calcium green-1 was microinjected into the root hairs. Under an Axiovert 200 M inverted microscope (Eppendorf TransferMan NK2, Hamburg, Germany), root hairs were impaled with micropipettes, pulled from filament electrode glass using a PC-84 puller (Sutter Instruments, Novato, CA, USA), that contained 0.5 mM dextran-conjugated dye. The dye was then pressure-injected into the root hair base on the microscope. The injected cells were allowed to recover for 30 min prior to imaging after the microinjection.

The long-wavelength calcium indicator Rhod-2 with net positive charge was used to measure [Ca^2+^]_m _in the root hairs. [Ca^2+^] was calculated according to the following formula: [Ca^2+^] = Kd * (F - F_min_)/(F_max _- F), where F is the fluorescence of the indicator at the experimental Ca^2+ ^levels, F_min _is the fluorescence in the absence of Ca^2+ ^and F_max _is the fluorescence of the Ca^2+^-saturated probe. In the Fluo-4 and Calcium green-1 labeling experiments, each single measurement of the F_max _value was determined by addition of 2 μM ionomycin and 5 mM Ca^2+^, and the F_min _value was determined by addition of 5 mM EGTA, where the Kd of Fluo-4 was 345 nM and that of Calcium green-1 was 190 nM [72,73]. In Rhod-2 labeling experiments, every measurement of the F_max _value was determined by addition of 2 μM ionomycin and 2 mM Mn^2+^, and the F_min _value was determined by addition of 5 mM EGTA, where the Kd of Rhod-2 was 570 nM [74].

### Drug treatments

All chemicals were purchased from Sigma (St. Louis, MO, USA) unless otherwise indicated. Stock concentrations of 1 mM Lat-B, 100 μM Jas, and 2 mM Cs A were prepared in DMSO. The working concentrations were as follows: 10-1000 nM Lat-B, 10-1000 nM Jas, and 2 μM Cs A. The working solutions were carefully added to the samples labeled with dye when taking photos. Cs A was added to the samples and treated 30 min after the dye loading, and fluorescence was collected after washout.

## Abbreviations

The abbreviations used are: MF: microfilament; Lat-B: latrunculin B; Jas: jasplakinolide; Cs A: cyclosporin A; [Ca^2+^]_c_: free cytoplasmic Ca^2+ ^concentration; [Ca^2+^]_m_: free Ca^2+ ^concentration in mitochondria; mPTP: mitochondrial permeability transition pore; F-actin: filamentous actin; G-actin: globular actin; ER: endoplasmic reticulum; Ψ_m_: mitochondrial membrane potential; VDAC: voltage-dependent anion channel; ANT: adenine nucleotide translocase; CICR: Ca^2+^-induced Ca^2+ ^release.

## Authors' contributions

YW and JL designed the study, YW and YZ carried out the experiments and the quantitative image analysis, and drafted the manuscript. YL carried out the experiment of cytosolic Ca^2+ ^labeling. HZ offered the guidance for experimental design. PL provided the data analysis and figures drawing supports. FB and JS offered the transgenic plant seeds and co-localization figure and analysis, and revised the manuscript. JL provided support and general guidance for this work. QW conceived of the study and results analysis, and guided for writing the manuscript. All authors read and approved the final manuscript.

## Supplementary Material

Additional file 1 **Movie 1. Time course of fluorescence changes in mitochondria labeled with Rhod-2 in the same root hair in 10 min duration of treatment with 500 nM Lat-B**. The movie was composed of a series of confocal option sections scanned at 50-s intervals. The first frame was the fluorescence image before treatment; others were the images after treatment. Click here for file

Additional file 2 **Figure S1. The [Ca^2+^]_c _gradient in a control root hair**. The root hair was micro-injected with Calcium green-1-dextran. Cytoplasmic calcium levels were pseudo-color-coded according to original intensity of green fluorescence. The bar on the right showed the relationship between Ca^2+ ^concentration and cellular pseudo-color. Scale bar = 10 μm. Click here for file

Additional file 3 **Figure S2. Changes in cytoplasmic Ca^2+ ^levels induced by Lat-B in the root hair**. (A) The pseudo-color image of the Ca^2+ ^concentration labeled with Fluo-4 in a normally growing root hair. Scale bar = 10 μm. (B-J) The pseudo-color images of the same root hair treated with 500 nM Lat-B in 360 s duration. The bar on the right showed the relationship between Ca^2+ ^concentration and cellular pseudo-color. Click here for file

Additional file 4 **Figure S3. Bright field image of illustrating the location of the scanning ion-selective electrodes in root hairs**. In the experiment of Ca^2+ ^flux measurement, the Ca^2+ ^selective probe positioned 2 μm from the tip of root hair surface to record the plasma membrane Ca^2+ ^fluxes. Scale bar = 10 μm. Click here for file
