# Supplementary figures and images for: Disruption of actin filaments induces mitochondrial Ca2+ release to the cytoplasm and [Ca2+]c changes in Arabidopsis root hairs
Source: BMC Plant Biol. 2010 Mar 24;10:53. doi: 10.1186/1471-2229-10-53 (PMC2923527; doi:10.1186/1471-2229-10-53)

**Figure 1**

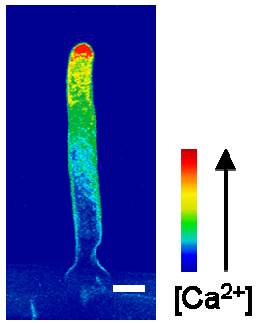

Supplement: Additional file 2 — Figure S1. The [Ca2+]c gradient in a control root hair. The root hair was micro-injected with Calcium green-1-dextran. Cytoplasmic calcium levels were pseudo-color-coded according to original intensity of green fluorescence. The bar on the right showed the relationship between Ca2+ concentration and cellular pseudo-color. Scale bar = 10 μm. [file 1471-2229-10-53-S2.PDF]

**Figure 2**

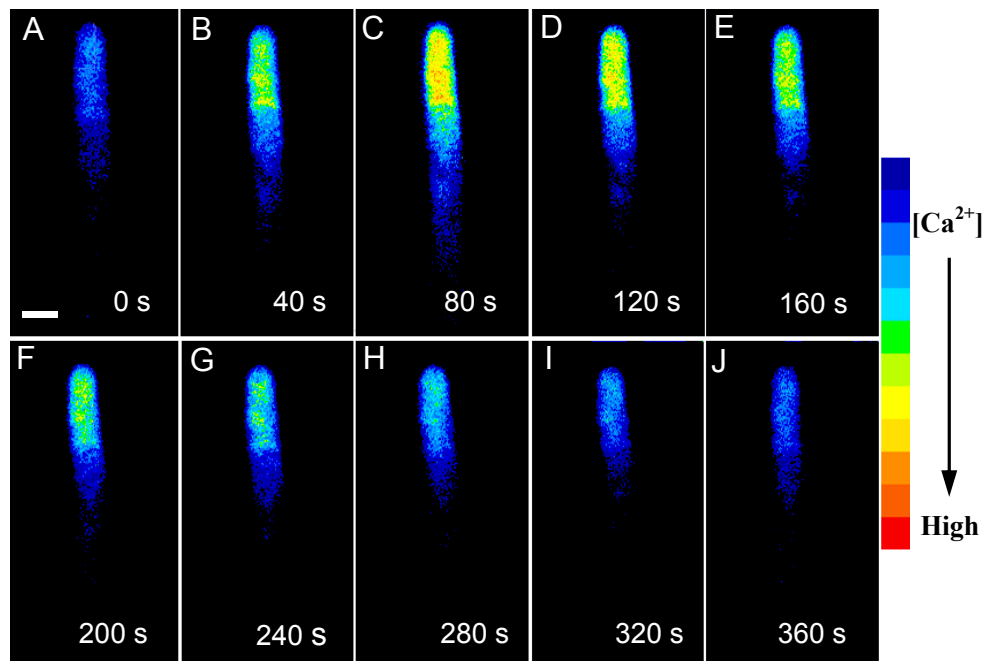

Supplement: Additional file 3 — Figure S2. Changes in cytoplasmic Ca2+ levels induced by Lat-B in the root hair. (A) The pseudo-color image of the Ca2+ concentration labeled with Fluo-4 in a normally growing root hair. Scale bar = 10 μm. (B-J) The pseudo-color images of the same root hair treated with 500 nM Lat-B in 360 s duration. The bar on the right showed the relationship between Ca2+ concentration and cellular pseudo-color. [file 1471-2229-10-53-S3.PDF]

**Figure 3**

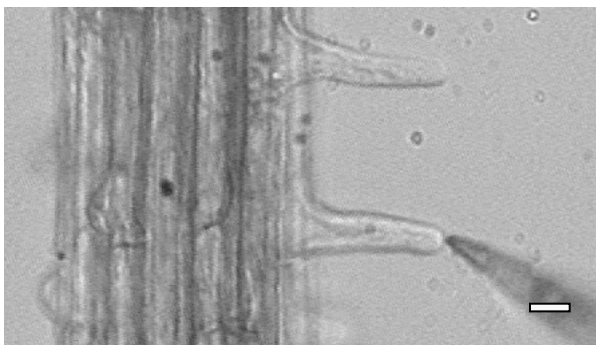

Supplement: Additional file 4 — Figure S3. Bright field image of illustrating the location of the scanning ion-selective electrodes in root hairs. In the experiment of Ca2+ flux measurement, the Ca2+ selective probe positioned 2 μm from the tip of root hair surface to record the plasma membrane Ca2+ fluxes. Scale bar = 10 μm. [file 1471-2229-10-53-S4.PDF]
